# Distinct sequential death complexes regulate pyroptosis and IL-1β release in response to *Yersinia* blockade of immune signaling

**DOI:** 10.1126/sciadv.adl3629

**Published:** 2024-07-26

**Authors:** Ronit Schwartz Wertman, Winslow Yost, Beatrice I. Herrmann, Christopher M. Bourne, Daniel Sorobetea, Christina K. Go, Benedikt S. Saller, Olaf Groß, Phillip Scott, Anthony Rongvaux, Cornelius Y. Taabazuing, Igor E. Brodsky

**Affiliations:** ^1^ Department of Pathobiology, University of Pennsylvania School of Veterinary Medicine, Philadelphia, PA 19104, USA.; ^2^ Department of Biochemistry and Biophysics, Perelman School of Medicine, University of Pennsylvania, Philadelphia, PA 19104, USA.; ^3^ Institute of Neuropathology, Medical Center - University of Freiburg, Faculty of Medicine, University of Freiburg, Freiburg 79106, Germany.; ^4^ Faculty of Biology, University of Freiburg, Freiburg 79106, Germany.; ^5^ Signalling Research Centres BIOSS and CIBSS, University of Freiburg, Freiburg 79106, Germany.; ^6^ Translational Science and Therapeutics Division, Fred Hutchinson Cancer Center, Seattle, WA 98109, USA.; ^7^ Department of Immunology, University of Washington, Seattle, WA 98195, USA.

## Abstract

Pathogen infection of host cells triggers an inflammatory cell death termed pyroptosis via activation of inflammatory caspases. However, blockade of immune signaling kinases by the *Yersinia* virulence factor YopJ triggers cell death involving both apoptotic caspase-8 and pyroptotic caspase-1. While caspase-1 is normally activated within inflammasomes, *Yersinia*-induced caspase-1 activation is independent of known inflammasome components. We report that caspase-8 is an essential initiator, while caspase-1 is an essential amplifier of its own activation through two feed-forward loops involving caspase-1 auto-processing and caspase-1–dependent activation of gasdermin D and NLPR3. Notably, while *Yersinia-*induced caspase-1 activation and cell death are inflammasome-independent, IL-1β release requires NLPR3 inflammasome activation. Mechanistically, caspase-8 is rapidly activated within multiple foci throughout the cell, followed by assembly of a canonical inflammasome speck, indicating that caspase-8 and canonical inflammasome complex assemblies are kinetically and spatially distinct. Our findings reveal that functionally interconnected but distinct death complexes mediate pyroptosis and IL-1β release in response to pathogen blockade of immune signaling.

## Teaser

*Yersinia* immune blockade drives cell death and IL-1β release via spatially distinct, functionally connected death complexes.

## INTRODUCTION

The innate immune system is critical for host defense against bacterial pathogens, as it detects pathogen-associated molecular patterns as well as pathogen-mediated perturbations of host biological pathways (*1*, *2*). Apoptosis, pyroptosis, and necroptosis are distinct forms of regulated cell death that mediate antimicrobial host defense (*3*, *4*). Apoptosis is classically viewed as a developmentally programmed or homeostatic, noninflammatory cell death, whereas pyroptosis is a lytic form of cell death accompanied by release of inflammatory interleukin-1 (IL-1) family cytokines that takes place in response to microbial infection (*5*–*7*). Apoptosis and pyroptosis are both driven through activation of caspases, cysteine protease zymogens that undergo proteolytic activation following recruitment to multiprotein complexes (*8*), while necroptosis is caspase-independent (*9*, *10*).

Apoptosis and pyroptosis require engagement of distinct signaling complexes and effector caspases and are traditionally thought to be mutually exclusive and cross-inhibitory (*3*). However, disruption of core immune signaling pathways by pathogen virulence factors can trigger cell death that exhibits biochemical features of both apoptosis and pyroptosis (*11*). Recent studies have proposed the existence of a cell death pathway involving simultaneous activation of pyroptosis, apoptosis, and necroptosis, termed PANoptosis (*12*, *13*), following microbial infection or disruption of immune signaling pathways. However, as the morphologic and physiologic consequences of distinct cell death pathways are unique, and the effector enzymes of one death pathway typically cross-inhibit the others, how an individual cell might simultaneously undergo multiple distinct forms of cell death is unclear.

During apoptosis, processing of executioner caspase-3 and caspase-7 by the initiator caspase-8 results in the cleavage of numerous caspase-3/7–dependent substrates, leading to the organized breakdown of the cell into membrane-enclosed blebs that are rapidly phagocytosed by neighboring cells with minimal inflammation (*5*). Conversely, during pyroptosis, caspase-1 is activated by its recruitment into inflammasomes, multiprotein signaling complexes that form in response to microbial contamination of the cytosol (*6*, *7*) and are nucleated by sensor NOD-like receptor (NLR) proteins and the adaptor protein ASC (apoptosis-associated speck–like protein containing a caspase activation and recruitment domain) (*14*). Active caspase-1 processes the inflammatory cytokine pro–IL-1β and pore-forming protein gasdermin D (GSDMD). The N-terminal fragment of GSDMD (p30) oligomerizes and inserts into the plasma membrane, releasing mature IL-1β as well as other intracellular alarmins through membrane rupture and cell lysis (*14*–*16*).

Pathogenic *Yersiniae* inject a variety of virulence factors known as *Yersinia* outer proteins (Yops) into the cytoplasm of host cells through their type 3 secretion systems (*17*–*19*) to disrupt innate immune responses. Among these is the acetyl-transferase YopJ, which blocks IκB kinase (IKK) and transforming growth factor β–activated kinase 1 (TAK1) signaling (*20*–*22*). Such blockade leads to the combined activation of caspase-1 and caspase-8 and elicits caspase-1–and caspase-8–dependent cleavage of GSDMD and IL-1β (*23*, *24*). Caspase-1 activation following *Yersinia pseudotuberculosis* (*Yptb*) infection is independent of all now known inflammasome components, including NLRP3, NLRC4, and the inflammasome adaptor protein ASC (*25*, *26*), but is instead dependent on caspase-8 (*25*, *26*). Despite the lack of a requirement for ASC in caspase-8 or caspase-1 activation or cell death (*25*), ASC forms large oligomers in response to YopJ activity, suggesting that ASC complexes play an as-yet-undefined role in *Yersinia* infection (*24*). While the ASC pyrin domain interacts with the caspase-8 death-effector domain (DED) (*24*, *27*, *28*), whether these distinct pathways are activated simultaneously or sequentially within infected cells and their role in promoting programmed cell death and inflammatory responses is poorly defined.

Here, we find that caspase-8–dependent caspase-1 activation requires both caspase-8 and caspase-1 activity. Unexpectedly, despite the ability of caspase-8 to cleave caspase-1 directly, caspase-1 catalytic activity was required for its own processing downstream of caspase-8 activation, indicating that caspase-1 acts as a feed-forward amplifier of caspase-8–dependent pyroptosis. Macrophages that express an uncleavable caspase-8 (*Casp8^D387A/D387A^*) are sensitized to receptor-interacting protein kinase 3 (RIPK3)–mediated necroptosis, which triggers a backup pathway of caspase-1 activation to enable pyroptosis and IL-1β release even in the absence of active caspase-8. In addition, although ASC is not required for caspase-1 activation or cytotoxicity during *Yptb* infection, IL-1β release requires the canonical NLRP3 inflammasome. These findings indicate that secondary NLRP3 inflammasome activation subsequent to GSDMD cleavage and potassium-efflux mediates IL-1β release during *Yptb *infection. Caspase-8 activation preceded assembly of ASC puncta, and ASC puncta and active caspase-8 were differentially localized within macrophages. Together, this work demonstrates that functionally linked but temporally and spatially distinct death complexes mediate pyroptosis and IL-1β release in response to blockade of innate immune signaling.

## RESULTS

### Caspase-8 activity is required for cell death and caspase-1 processing

**Fig. 1.**
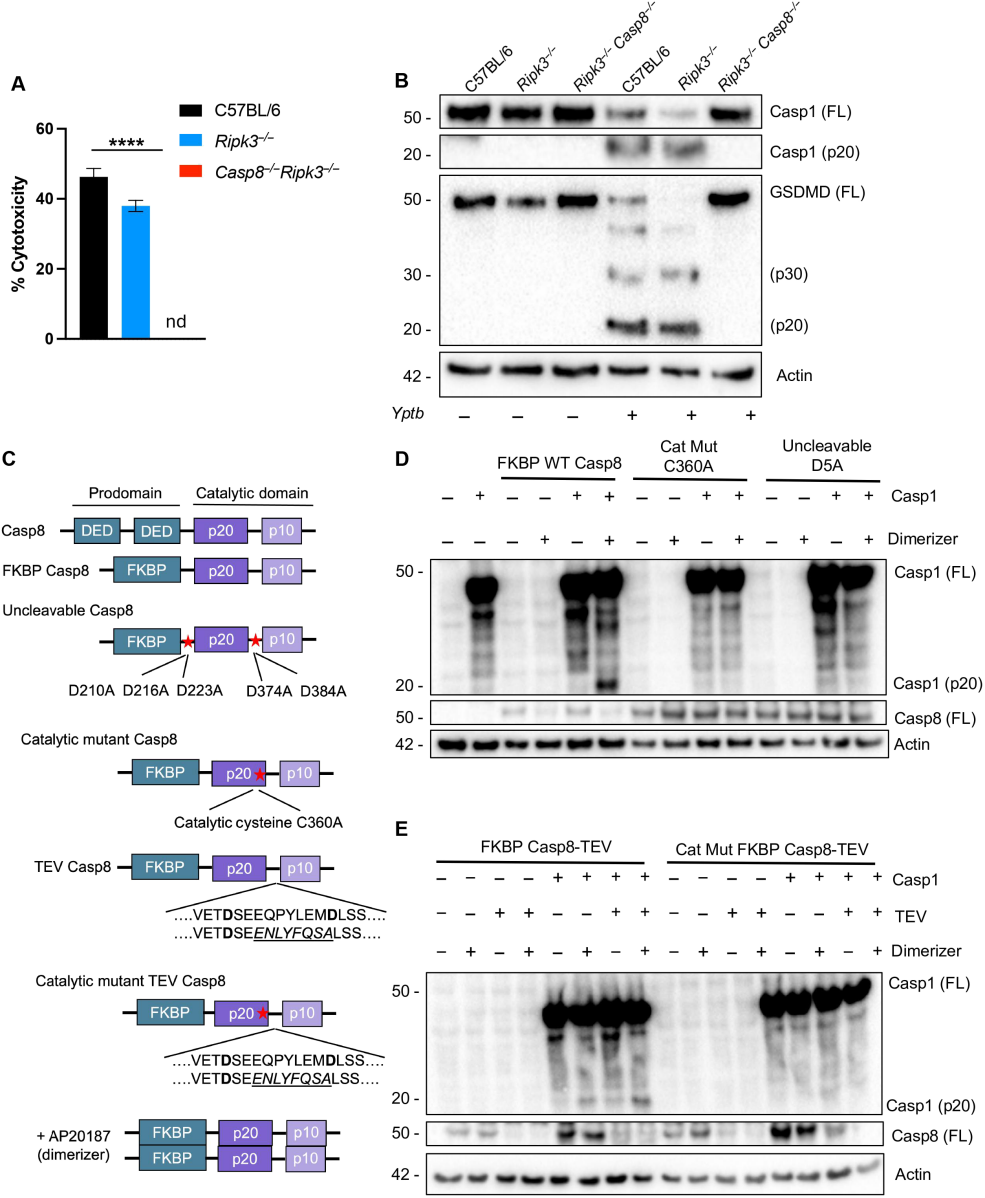
Caspase-8 activity is required for cell death and caspase-1 processing. (**A**) C57BL/6, *Ripk3^−/−^*, and *Casp8^−/−^Ripk3^−/−^* bone marrow–derived macrophages (BMDMs) were infected with wild-type (WT) *Yptb* and percent cytotoxicity was measured 4 hours after infection as described in Materials and Methods. (**B**) Lysates collected 3 hours after infection were immunoblotted for caspase-1 and GSDMD. FL indicates full-length protein. (**C**) Schematic representation of FKBP constructs of caspase-8 used in this study. (**D**) Human embryonic kidney (HEK) 293T cells transfected with caspase-1 and WT, catalytically inactive (C360A), or uncleavable (D5A) FKBP–caspase-8 and induced to dimerize with AP20187 (dimerizer) 24 hours after transfection. Lysates were collected for Western blotting 6 hours after adding AP20187. (**E**) HEK293T cells transfected with caspase-1, tobacco etch virus (TEV), and WT or catalytically inactive (C360A) FKBP–caspase-8–TEV and treated with dimerizer as indicated 24 hours after transfection. Lysates were collected for Western blotting 6 hours after AP20187 addition. nd, not detected; ****P < 0.0001 by two-way analysis of variance (ANOVA). Error bars represent the means ± SEM of triplicate wells and are representative of three independent experiments.

During *Yptb* infection, cell death, and caspase-1 processing occur independently of all known inflammasome components (*25*). Consistent with previous findings from our group and others (*25*, *29*), cell death in response to *Yersinia* YopJ activity is dependent on caspase-8, as in contrast to either *Ripk3^−/−^* or C57BL/6 bone marrow–derived macrophages (BMDMs), *Casp8^−/−^Ripk3^−/−^* BMDMs remain viable following *Yptb* infection (Fig. 1A). Furthermore, processing of caspase-1 into its active p20 fragment is dependent on caspase-8, as, unlike in *Ripk3^−/−^* BMDMs, it is not observed in *Casp8^−/−^Ripk3^−/−^* BMDMs (Fig. 1B) (*25*, *29*). Consistent with prior findings (*23*, *24*, *30*), GSDMD processing also requires caspase-8 but not RIPK3 (Fig. 1B). Because of the embryonic lethality of caspase-8 single-knockout mice (*9*, *10*), *Casp8^−/−^* BMDMs cannot be used as a control. To determine whether caspase-8 is sufficient for caspase-1 activation, as well as to define its molecular requirements, we co-expressed caspase-1 with various caspase-8 constructs in which the caspase-8 DEDs were replaced with an inducible dimerizable domain that promotes its activation upon addition of the dimerizer compound AP20187 (Fig. 1C) (*31*). Addition of AP20187 to induce caspase-8 dimerization triggered robust caspase-1 processing into its active p20 fragment, which was undetectable in the absence of dimerizer (Fig. 1D). Critically, dimerizable constructs containing catalytic mutant caspase-8 (C360A) or uncleavable caspase-8^31^ were unable to mediate caspase-1 cleavage, indicating that caspase-8–dependent caspase-1 cleavage requires both caspase-8 catalytic activity and auto-processing (Fig. 1D). To determine whether auto-processed caspase-8 functions solely as a scaffold to recruit caspase-1 or whether its catalytic activity is directly necessary for caspase-1 processing, we expressed dimerizable caspase-8 constructs in which the interdomain auto-processing site at position D384 was replaced with the cleavage sequence for the tobacco etch virus (TEV) protease. While addition of dimerizer to cells co-transfected with caspase-8–TEV and caspase-1 led to some baseline caspase-1 processing, inducing caspase-8–TEV cleavage by co-expression of TEV protease resulted in robust caspase-1 processing (Fig. 1E). Caspase-8 catalytic activity was essential for caspase-1 activation even when caspase-8 was dimerized and exogenously cleaved by TEV, demonstrating that both caspase-8 cleavage and enzymatic activity are required for caspase-1 processing (Fig. 1E).

### Caspase-1 activation by caspase-8 requires caspase-1 catalytic activity

**Fig. 2.**
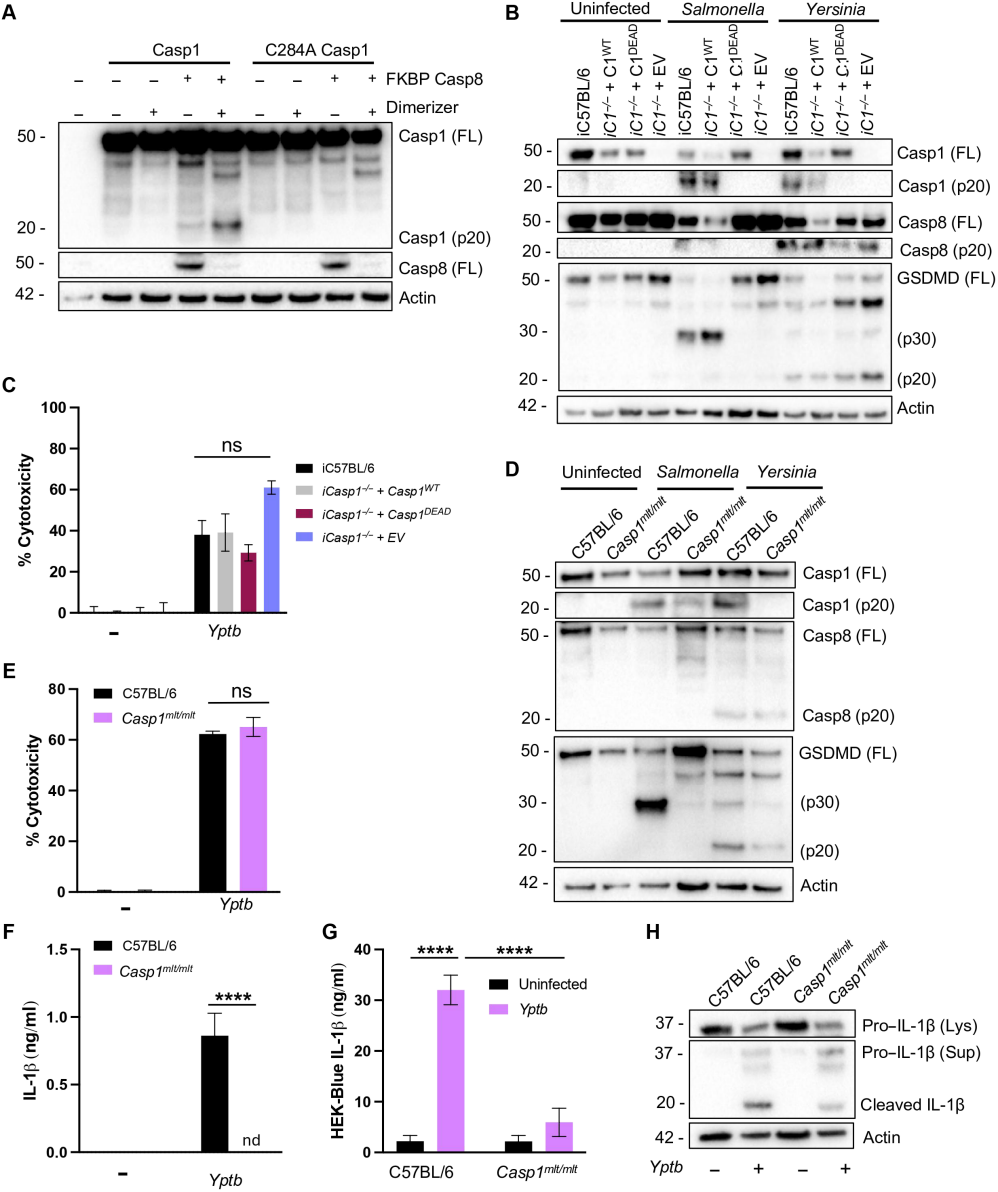
Caspase-1 activation by caspase-8 requires caspase-1 catalytic activity. (**A**) HEK293T cells were transfected with FKBP–caspase-8 and WT or catalytically inactive (C284A) caspase-1 and treated with dimerizer. Lysates were collected for Western blotting as described in Materials and Methods. FL indicates full-length protein. (**B**) iC57BL/6, *iCasp1^−/−^ + Casp1^WT^*, *iCasp1^−/−^ + Casp1^DEAD^*, and *iCasp1^−/−^ + Empty Vector* immortalized BMDMs (iBMDMs) were infected with WT *Yptb* as described in Materials and Methods. Lysates collected 3 hours after infection were immunoblotted for caspase-1, caspase-8, GSDMD, and β-actin as indicated. (**C**) Percent cytotoxicity was assayed 4 hours after infection as described in Materials and Methods. (**D**) C57BL/6 and *Casp1^mlt/mlt^* BMDMs were infected with WT *Yptb* as described. Lysates collected 3 hours after infection were immunoblotted for caspase-1, caspase-8, GSDMD, and β-actin. (**E**) Percent cytotoxicity was measured 4 hours after infection. (**F**) Release of IL-1β into the supernatant was measured by enzyme-linked immunosorbent assay (ELISA) 4 hours after infection. (**G**) Release of IL-1β into the supernatant was measured by HEK-Blue IL-1R reporter assay 4 hours after infection. (**H**) Lysates and supernatants collected 3 hours after infection were immunoblotted for IL-1β. ns, not significant; ****P < 0.0001 by two-way ANOVA. Error bars represent the means ± SEM of triplicate wells and are representative of three independent experiments.

Our findings indicate that caspase-8 acts as an apical initiator that activates caspase-1 in response to blockade of TAK1 and IKK signaling by pathogens. Caspase-1 autoproteolysis is required for its activation within canonical inflammasomes (*32*–*34*). In contrast, during apoptosis, caspase-8 processes caspase-3 into its mature form, but caspase-3 does not undergo autoproteolysis, thus limiting its feed-forward amplification capacity (*34*, *35*). To test whether caspase-1 catalytic activity is required for full caspase-1 activation following the initial activation by caspase-8, we activated FK506 binding protein (FKBP)–caspase-8 in human embryonic kidney (HEK) 293T cells in the presence of wild-type (WT) or catalytically inactive caspase-1. Unexpectedly, catalytically inactive caspase-1 (Casp1^C284A^) failed to undergo processing in response to inducible dimerization of caspase-8, indicating that caspase-1 catalytic activity is required for its own processing in a feed-forward manner following its cleavage by caspase-8 (Fig. 2A). Consistently, while *Casp1^−/−^* immortalized BMDMs (iBMDMs) stably expressing WT caspase-1 robustly processed caspase-1 upon infection with *Salmonella Typhimurium* or *Yptb* (Fig. 2B), iBMDMs expressing catalytically inactive caspase-1, known as caspase-1 DEAD (C284A) (*33*) were unable to process caspase-1 during either *Salmonella* or *Yptb* infection. These observations support our findings that caspase-1 catalytic activity is necessary for its own processing and activity downstream of YopJ-induced caspase-8 activation (Fig. 2B). Consistent with previous findings that *Yptb-*induced cell death does not require caspase-1 or caspase-11 (*24*, *25*), caspase-1 DEAD cells exhibited WT levels of lactate dehydrogenase (LDH) release upon infection with *Yptb* (Fig. 2C) but failed to induce LDH release upon infection with *S. Typhimurium*, (Fig. 2C and fig. S1A). Critically, primary BMDMs from knock-in mice lacking caspase-1 catalytic activity (*Casp1^mlt/mlt^*) (*36*) also failed to process caspase-1 and had significantly reduced processing of GSDMD (Fig. 2D). Similarly to the C284A iBMDMs, cytotoxicity responses in *Casp1^mlt/mlt^* BMDMs to *Yptb* infection were normal, despite these cells being unable to undergo cytotoxicity in response to *Salmonella* (Fig. 2E and fig. S1B). Moreover, IL-1β processing and release were also reduced in response to *Yptb* infection in *Casp1^mlt/mlt^* BMDMs (Fig. 2, F to H). In contrast to IL-1β release, IL-12 secretion by *Casp1^mlt/mlt^* BMDMs was largely intact (fig. S1C), and caspase-3 and GSDME cleavage were unaffected (fig. S1D). Together, our results demonstrate that caspase-8 and caspase-1 enzymatic activities are both critical for caspase-1 processing in response to *Yptb* infection and that caspase-1 catalytic activity is required for IL-1β secretion, even in the presence of sufficient caspase-8 activity.

### Caspase-8 auto-processing limits RIPK3-mediated necroptosis

**Fig. 3.**
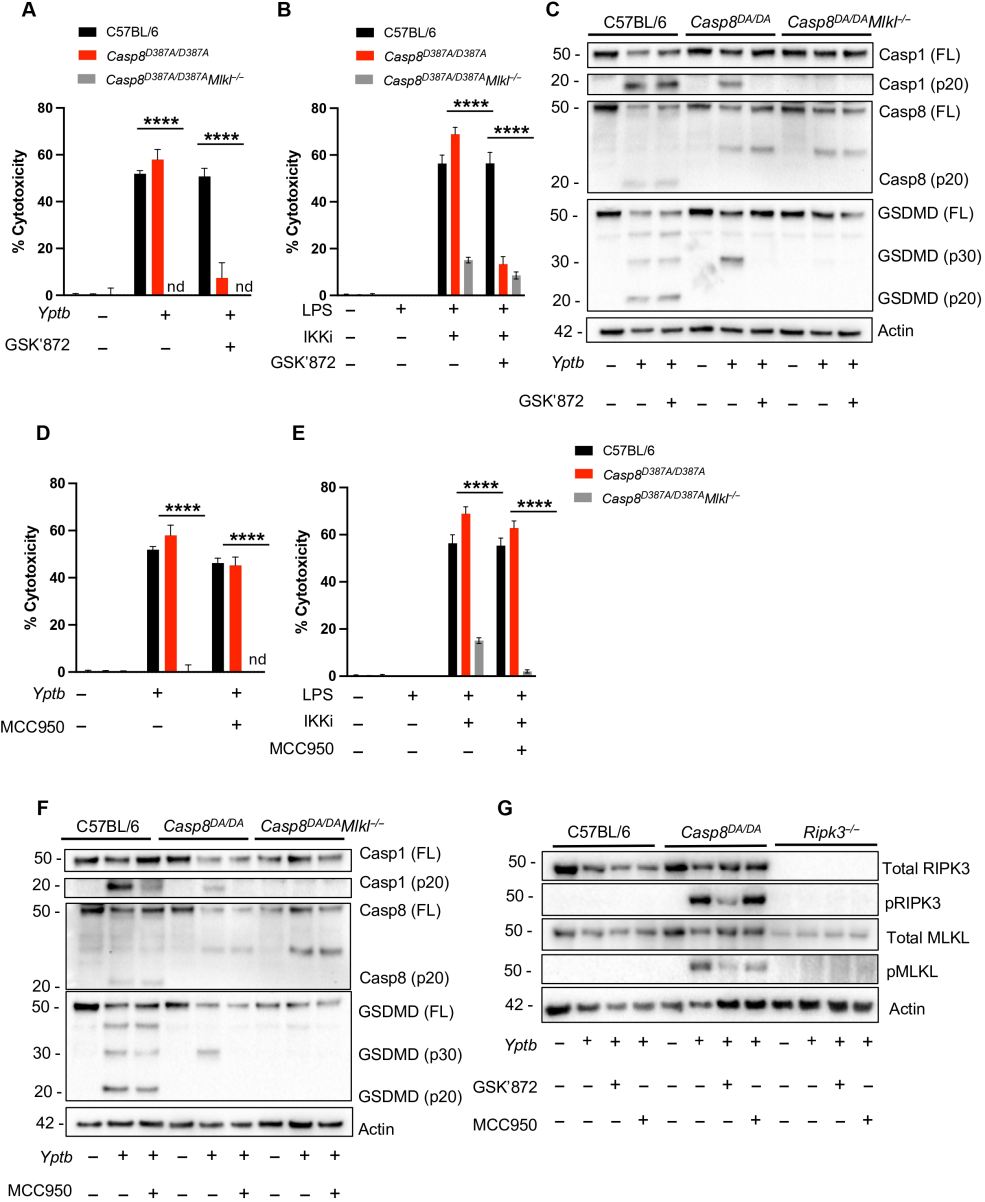
Caspase-8 auto-processing limits RIPK3-mediated necroptosis. (**A**) C57BL/6, *Casp8^D387A/D387A^*, and *Casp8^D387A/D387A^Mlkl^−/−^* BMDMs were treated with GSK′872 or vehicle control as indicated and infected with WT *Yptb*. Percent cytotoxicity was measured 4 hours after infection as described. (**B**) C57BL/6, *Casp8^D387A/D387A^*, and *Casp8^D387A/D387A^Mlkl^−/−^* BMDMs were primed with LPS followed by IKK inhibitor (IKKi). Before IKKi treatment, BMDMs were treated with GSK′872 or vehicle control. Percent cytotoxicity was measured 5 hours after infection. (**C**) Lysates collected 3 hours after infection were immunoblotted for caspase-1, caspase-8, GSDMD, and β-actin. FL indicates full-length protein. (**D**) C57BL/6, *Casp8^D387A/D387A^*, and *Casp8^D387A/D387A^Mlkl^−/−^* BMDMs were treated with MCC950 or vehicle control and were infected with WT *Yptb*. Percent cytotoxicity was measured 4 hours after infection. (**E**) C57BL/6, *Casp8^D387A/D387A^*, and *Casp8^D387A/D387A^Mlkl^−/−^* BMDMs were primed with LPS followed by IKKi. Before IKKi treatment, BMDMs were treated with MCC950 or vehicle control. Percent cytotoxicity was measured 5 hours after infection. (**F**) Lysates collected 3 hours after infection were immunoblotted for caspase-1, caspase-8, GSDMD, and β-actin. (**G**) C57BL/6, *Casp8^D387A/D387A^*, and *Ripk3^−/−^* BMDMs were treated with MCC950, GSK′872, or vehicle control and were infected with WT *Yptb*. Lysates collected 3 hours after infection were immunoblotted for total RIPK3, pRIPK3, total MLKL, pMLKL, and β-actin. ****P < 0.0001 by two-way ANOVA. Error bars represent the means ± SEM of triplicate wells and are representative of three independent experiments.

*Yersinia* infection or TAK1 blockade has been proposed to induce a combined form of cell death termed PANoptosis, involving the simultaneous activation of pyroptosis, apoptosis, and necroptosis, as assessed by phosphorylation of RIPK3 and the mixed lineage kinase domain–like pseudokinase (MLKL) pore-forming protein, coincident with activation of apoptotic and pyroptotic caspases (*12*, *13*, *37*). However, in the absence of caspase-8 auto-processing, cells undergo RIPK3-dependent necroptosis mediated by RIPK3-dependent activation of MLKL (*38*, *39*). Because MLKL pore formation can promote potassium efflux, a common trigger of the NLRP3 inflammasome (*38*, *40*, *41*), we hypothesized that caspase-1 activation in the absence of caspase-8 auto-processing could result from NLRP3 activation downstream of RIPK3- and MLKL-mediated necroptosis. Therefore, we assayed cell death and caspase-1 processing in *Casp8^D387A/D387A^* BMDMs, which express an uncleavable caspase-8, either after infection with *Yptb* or treatment with lipopolysaccharide (LPS)/IKK inhibitor (IKKi), which pharmacologically mimics the activity of YopJ (*42*). In contrast to the HEK293T system or in our previous studies in which non-cleavable caspase-8 was expressed in cells lacking RIPK3 (*25*), we found that *Casp8^D387A/D387A^* BMDMs infected with *Yptb* or treated with LPS/IKKi exhibited comparable LDH release and caspase-1 processing as WT BMDMs (Fig. 3, A to C). Moreover, in *Casp8^D387A/D387A^* BMDMs, *Yptb* infection induced processing of GSDMD only into the active p30 fragment, whereas WT BMDMs processed GSDMD into both p30 and p20 fragments (Fig. 3C). The GSDMD p20 fragment is generated by caspase-3–mediated cleavage (*43*), indicating that both caspase-1 and caspase-3 are active in WT macrophages, but only caspase-1 is active in *Casp8^D387A/D387A^* macrophages. Moreover, the RIPK3 inhibitor GSK′872 inhibited both cell lysis and caspase-1 processing in *Casp8^D387A/D387A^* but not WT BMDMs following *Yptb* infection or LPS/IKKi treatment, suggesting that caspase-8 auto-processing during *Yersinia* infection or IKK/TAK1 blockade normally limits RIPK3-mediated necroptosis and subsequent caspase-1 activation (Fig. 3, A to C). Notably, while the NLRP3-specific inhibitor MCC950 did not inhibit cell lysis, it completely blocked caspase-1 and GSDMD processing in *Yptb-*infected *Casp8^D387A/D387A^* BMDMs, indicating that caspase-1 processing downstream of RIPK3 activation is mediated by NLRP3 (Fig. 3, D to F). *Casp8^D387A/D387A^Mlkl^−/−^* BMDMs (*44*) exhibited no cell lysis, caspase-1, caspase-8, or GSDMD processing, indicating that MLKL activation occurs upstream of caspase-1 activation, GSDMD processing, and cell lysis in *Casp8^D387A/D387A^* BMDMs (Fig. 3, A to F). In addition, caspase-8 auto-processing was required for caspase-3 activation and subsequent cleavage of GSDME (fig. S2A). RIPK3 and MLKL were not phosphorylated in WT BMDMs following *Yptb* infection, indicating that the necroptosis pathway is not cryptically activated during *Yptb* infection or IKK blockade (Fig. 3G). This observation is consistent with the lack of requirement for RIPK3 or MLKL in *Yptb-*induced death of BMDMs (*25*) but contrasts with the reported phosphorylation of RIPK3 and MLKL during PANoptosis (*12*, *13*, *37*). Instead, our findings indicate that caspase-8 auto-processing directly activates caspase-1 and that, in caspase-8 auto-processing-deficient BMDMs, a backup caspase-1 activation pathway occurs via RIPK3- and MLKL-dependent activation of NLRP3.

To directly test the model that in the absence of caspase-8 auto-processing, NLRP3 inflammasome activation takes place downstream of necroptosis in a cell-intrinsic manner, we differentially labeled and cocultured *Casp8^D387A/D397A^* and *Casp8^D387A/D387A^Mlkl^−/−^* BMDMs, which can or cannot undergo necroptosis in response to *Yptb*, respectively (fig. S2, B to D). Critically, we observed that, in an infected coculture of both genotypes, only *Casp8^D387A/D397A^* cells underwent cell death in response to *Yptb*, as none of the CellTracker-labeled cells were co-stained with the Live/Dead dye (fig. S2, C and D). As expected, solo-cultured *Casp8^D387A/D397A^* BMDMs underwent cell death, while *Casp8^D387A/D387A^Mlkl^−/−^* cells did not (fig. S2, C and D). As a control, we observed that CellTracker-labeled *Casp8^D387A/D397A^* cells underwent cell death (fig. S2E), ruling out the possibility that the Live/Dead dye was diffusing out of CellTracker-labeled cells. These studies demonstrate that necroptosis in one cell does not signal neighboring cells to undergo cell death, indicating that NLRP3 inflammasome activation occurs in a cell intrinsic manner downstream of MLKL pore formation when Casp8 cannot undergo auto-processing.

### ASC speck formation is GSDMD- and NLRP3-dependent and is required for IL-1β release

**Fig. 4.**
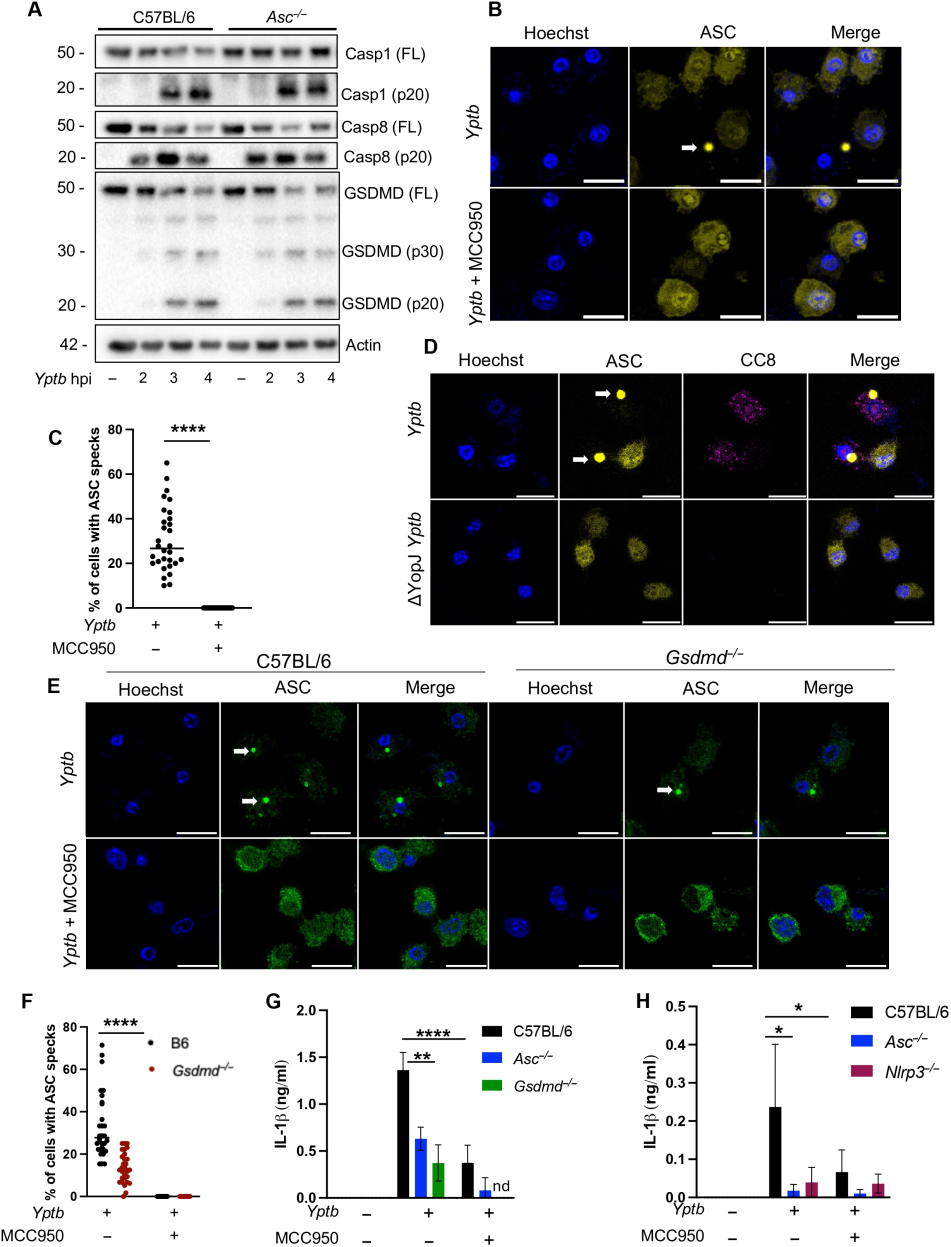
ASC speck formation is GSDMD and NLRP3 dependent and is required for IL-1β release. (**A**) C57BL/6, and *Asc^−/−^* BMDMs were infected with WT *Yptb*. Lysates collected 3 hours after infection were immunoblotted for caspase-1, caspase-8, GSDMD, and β-actin. hpi, hours postinfection. FL indicates full-length protein. (**B**) C57BL/6 ASC-citrine BMDMs were treated with MCC950 or vehicle control and were infected with WT *Yptb*. ASC speck formation was analyzed at 4 hours after infection. (**C**) Percent of cells with ASC specks was quantified. (**D**) C57BL/6 ASC-citrine BMDMs were infected with WT and Δ*yopJ Yptb*. Caspase-8 cleavage and ASC speck formation were analyzed at 4 hours after infection. (**E**) C57BL/6 and *Gsdmd^−/−^* BMDMs were treated with MCC950 or vehicle control and infected with WT *Yptb*. ASC speck formation was analyzed at 4 hours after infection via immunofluorescence staining. (**F**) Percent of cells with ASC specks was quantified. (**G**) Release of IL-1β into the supernatant was measured by ELISA at 4 hours after infection in C57BL/6, and *Asc^−/−^*, *Gsdmd^−/−^* BMDMs. (**H**) Release of IL-1β into the supernatant was measured by ELISA at 4 hours after infection in C57BL/6 and *Asc^−/−^*, *Nlrp3^−/−^* BMDMs. *P < 0.01, **P < 0.001 and ****P < 0.0001 by two-way ANOVA. Error bars represent the means ± SEM of triplicate wells and are representative of three independent experiments.

While our findings indicate that NLRP3 activates caspase-1 downstream of RIPK3/MLKL when caspase-8 activation is disrupted, whether and how NLRP3 might contribute to anti-*Yptb* responses in WT BMDMs is unclear. Although the NLRP3 inflammasome is activated in response to *Yersinia* infection or IKK/TAK1 blockade (*24*, *45*), NLRP3 and the adaptor ASC do not contribute to either caspase-8 or caspase-1 activation or cytotoxicity (*25*, *26*). It has been suggested that co-assembly of ASC, NLRP3, RIPK3, caspase-1, and caspase-8 triggers PANoptosis during *Yersinia* infection (*12*). However, activation of GSDMD can lead to formation of pores that mediate potassium efflux, a common trigger of the NLRP3 inflammasome that can promote feed-forward activation of caspase-1 downstream of other stimuli (*46*–*48*). Intriguingly, while we did not observe any differences between WT and *Asc^−/−^* BMDMs in the extent or kinetics of caspase-1, caspase-8, or GSDMD processing, robust caspase-8 processing occurred substantially earlier than processing of either caspase-1 or GSDMD (Fig. 4A), consistent with a model in which caspase-8 activation occurs upstream of NLRP3-dependent caspase-1 activation.

To determine whether NLRP3 inflammasome activation occurs downstream of GSDMD pore formation following *Yptb* infection, we assessed NLRP3 activation by the formation of large ASC specks that can be visualized via fluorescence microscopy (fig. S3A) (*49*). Transgenic BMDMs expressing ASC-citrine (*50*) exhibited robust formation of ASC specks following *Yptb* infection (Fig. 4, B to D, and fig. S3B). The NLRP3-specific inhibitor MCC950 abrogated ASC speck formation (Fig. 4, B and C), indicating that ASC speck assembly is NLRP3-dependent. During *Yersinia* infection, caspase-8 is activated at endosomal membranes by recruitment to RAGulator complexes (*51*). Both caspase-8 activation and ASC speck formation were dependent on YopJ activity (Fig. 4D). Cytotoxicity remained unchanged in infected WT and *Asc^−/−^* BMDMs even with MCC950 treatment, and we observed a slight but nonsignificant reduction in cytotoxicity in *Nlrp3^−/−^* BMDMs, suggesting that NLRP3 inflammasome activation and ASC speck formation occur downstream of GSDMD pore formation and induction of cell lysis following *Yptb* infection (fig. S3C). Consistently, *Gsdmd^−/−^* BMDMs had significantly fewer ASC specks relative to WT cells in response to *Yptb* infection (Fig. 4, E and F). Furthermore, MCC950 treatment or loss of ASC, GSDMD, or NLRP3 significantly reduced IL-1β release in response to *Yptb* infection (Fig. 4, G and H). Pannexin-1 is an apoptotic channel involved in NLRP3 inflammasome activation downstream of caspase-3–dependent cleavage (*43*); however, Pannexin-1 deficiency did not affect cell death or IL-1β release following *Yptb* infection (fig. S3, D and E). Furthermore, caspase-3 and GSDME cleavage were unaffected in *Asc^−/−^* BMDMs (fig. S3F), and combined loss of caspase-3 and caspase-7 did not affect cell death during *Yptb* infection (fig. S3G). Together, our results show that caspase-8–and caspase-1–mediated pyroptosis can occur in the absence of apoptotic caspases and that NLRP3 inflammasome activation downstream of caspase-8–and caspase-1–dependent GSDMD pore formation mediates ASC oligomerization and IL-1β release.

### Caspase-8 and ASC form separate but functionally linked death complexes

**Fig. 5.**
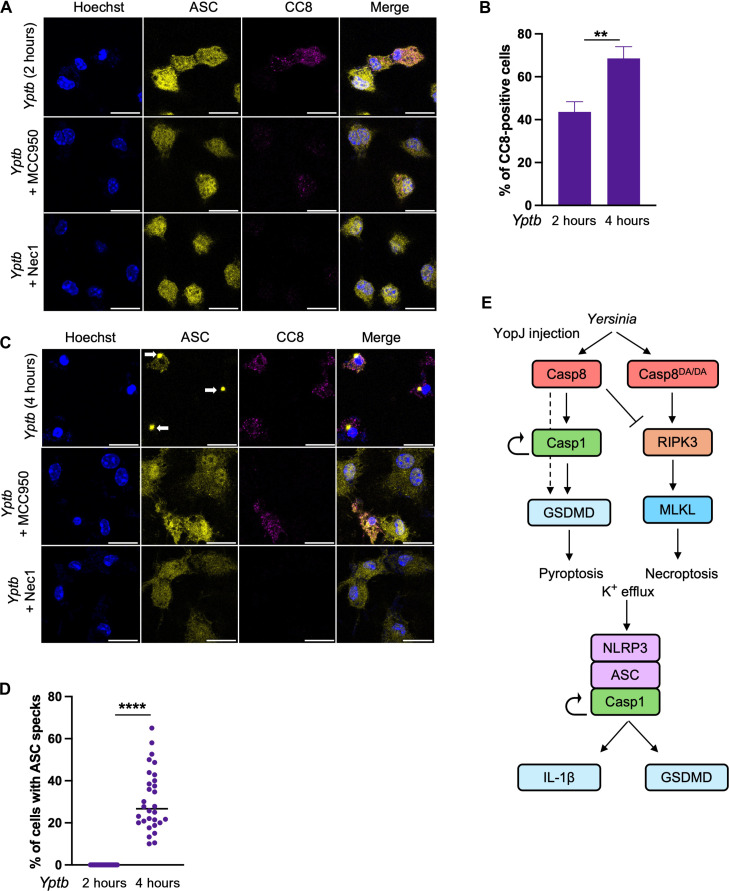
Caspase-8 and ASC form separate but functionally linked death complexes. (**A**) C57BL/6 ASC-citrine BMDMs were treated with MCC950, Nec-1, or vehicle control and were infected with WT *Yptb*. Caspase-8 cleavage and ASC speck formation were analyzed at 2 hours after infection. (**B**) Percent of cleaved caspase-8 (CC8)–positive cells was quantified at 2- and 4 hours after infection. (**C**) C57BL/6 ASC-citrine BMDMs were treated with MCC950, Nec-1, or vehicle control and were infected with WT *Yptb*. Caspase-8 cleavage and ASC speck formation were analyzed at 4 hours after infection. (**D**) Quantification of percent of cells with ASC specks at 2- and 4 hours after infection. (**E**) Graphical model: *Yersinia* YopJ induces caspase-8–dependent caspase-1 activation, which also requires caspase-1 catalytic activity and autoprocessing (circular arrow). Caspase-8–and caspase-1–dependent activation of GSDMD promotes canonical NLRP3- and ASC-dependent inflammasome activation that further amplifies GSDMD cleavage and mediates IL-1β release. When caspase-8 autoprocessing is unable to take place, RIPK3- and MLKL-dependent necroptosis leads to potassium efflux-induced activation of the canonical NLRP3- and ASC-dependent inflammasome activation that also mediates GSDMD cleavage and IL-1β release. ****P < 0.0001 by two-way ANOVA and **P < 0.05 by unpaired *t* test. Error bars represent the means ± SEM of triplicate wells and are representative of three independent experiments.

Our observations that ASC speck formation occurs downstream of GSDMD and is NLRP3-dependent, and that caspase-8 processing precedes caspase-1 processing suggest that, rather than simultaneous engagement of multiple death pathways within a single complex, *Yptb* infection induces sequential activation of distinct death complexes. Whereas robust caspase-8 activation was detected as early as 2 hours after infection and increased by 4 hours, ASC specks were undetectable at 2 hours and were only observed 4 hours after infection (Fig. 5, A to D, and fig. S4A). In addition to the observation that activation of caspase-8 and formation of ASC puncta are temporally distinct, we also observed virtually no colocalization between active caspase-8 puncta and the ASC speck (Fig. 5C). Moreover, both caspase-8 activity and ASC speck formation were abrogated upon treatment with the RIPK1 kinase inhibitor Nec-1, whereas the NLRP3 inhibitor MCC950 abrogated ASC speck formation but not active caspase-8 puncta formation (Fig. 5, A and C). Collectively, these data indicate that ASC speck formation occurs downstream of caspase-8 activation and is dependent on NLRP3. As both caspase-8 and caspase-1 cleavage of GSDMD can promote NLRP3 activation and ASC speck formation, whether caspase-8 is sufficient, in the absence of caspase-1, to fully activate the NLRP3 inflammasome is unclear. Notably, *Casp1/11^−/−^* ASC-citrine BMDMs exhibited a significant decrease in ASC specks compared to WT ASC-citrine BMDMs in responses to *Yptb* infection but not in response to LPS/adenosine 5′-triphosphate (ATP) (fig. S4, B to D). These data support a model whereby ASC speck formation is downstream of caspase-8–dependent caspase-1 activation in response to YopJ-dependent blockade of immune signaling but upstream of caspase-1 activation in response to canonical inflammasome stimuli such as LPS/ATP. Together, these data show that, in response to blockade of immune signaling due to *Yptb* infection, active caspase-8 and ASC complex assembly occur in a kinetically and spatially separable manner.

## DISCUSSION

The activation of pyroptotic and apoptotic caspases, along with the activation of necroptosis when caspase-8 is absent or inhibited during a variety of microbial infections or challenges, has led to a proposed model in which a single complex containing regulators of multiple death pathways (pyroptosis, apoptosis, and necroptosis) mediates *Yersinia-* and TAK1 blockade–induced cell death (*12*,*13*). Our findings support an alternative model in which spatially and temporally distinct yet functionally linked death complexes assemble in response to *Yptb* infection. Overall, our data indicate that upon *Yptb *infection or TAK1/IKK blockade, caspase-8 initiates downstream responses via direct cleavage of caspase-1, followed by auto-amplification of caspase-1 activation. Caspase-1 activation in response to *Yersinia* infection requires Fas-associated death domain protein (FADD) and RIPK1 (*25*), and the formation of the FADD/RIPK1/caspase-8–containing complex IIa downstream of TAK1 inactivation (*26*, *52*) suggests that caspase-1 activation initially takes place within this complex (*53*–*55*). Caspase-1 and caspase-8 activation within complex IIa also mediates GSDMD cleavage, for which our findings suggest that caspase-1 serves as the primary activator. Our data further demonstrate that GSDMD-dependent activation of the canonical NLRP3–ASC–caspase-1 inflammasome, presumably via potassium efflux, is kinetically and spatially distinct from caspase-8 activation and is not required for cell death but rather mediates the secretion of IL-1β. Thus, while caspase-8 can cleave GSDMD to induce pyroptosis in the absence of caspase-1, caspase-8 cannot compensate for lack of caspase-1 or NLRP3 inflammasome activation with respect to IL-1β secretion. Why caspase-1 and the NLRP3 inflammasome are required for IL-1β secretion despite upstream activation of caspase-8 is not clear but may be due to relative differences in substrate preference of caspase-8 and caspase-1 for IL-1β, efficiency of IL-1β recruitment to the inflammasome relative to complex IIa, and efficiency of caspase-1 versus caspase-8 recruitment to the NLRP3 inflammasome.

As *Yptb* infection induces caspase-1 cleavage and activation in the absence of known inflammasome components (*25*, *26*), we hypothesized that caspase-8 might directly activate caspase-1. Caspase-8 was sufficient, in a HEK293 expression system to induce caspase-1 processing in a manner requiring caspase-8 auto-processing and catalytic activity. Unexpectedly, we found that caspase-1 catalytic activity was also required for its own processing and activation downstream of IKK blockade. Our data suggest that the enzymatic activity of caspase-8 is required to generate a catalytically active scaffold, which can then recruit and cleave caspase-1. Our data further indicate that caspase-1 activity is required for its own processing, which likely occurs first within complex IIa and, subsequently, within NLRP3 inflammasomes, thereby amplifying the response to enable maximal cleavage of GSDMD, IL-1β, and pyroptosis.

Consistent with prior findings that caspase-1 and caspase-11 are not required for death of BMDMs in response to *Yersinia* (*25*), caspase-1 catalytic activity is dispensable for cell death, likely due to caspase-8–dependent cleavage of caspase-3 and caspase-7. GSDME, which is activated by caspase-3 and mediates pyroptosis in other settings (*56*–*58*), does not contribute to cell death during *Yersinia* infection (*30*), indicating that other caspase-3/7 targets are likely responsible. In addition, consistent with previous reports (*23*, *24*), in the absence of caspase-1, caspase-8–dependent cleavage of GSDMD also occurs and contributes to pyroptosis, although GSDMD cleavage is significantly blunted in the absence of caspase-1.

Simultaneous activation of multiple cell death pathways involving RIPK3 and caspase-8/caspase-1 is proposed to occur during infection by a number of pathogens including *Legionella*, *Francisella*, influenza, and *Yersinia* (*11*, *59*–*61*). How such a complex assembles remains mysterious, particularly when caspase-8 activity represses RIPK3-dependent necroptosis (*9*, *10*). RIPK3 makes no detectable contribution to *Yersinia-*induced cell death (*25*, *29*, *62*), and we do not observe any evidence for RIPK3-mediated necroptosis during *Yersinia* infection in the presence of functional caspase-8. Moreover, neither RIPK3 nor MLKL undergoes phosphorylation in WT cells during *Yersinia* infection or IKK blockade, suggesting that necroptosis is not activated by *Yersinia* when caspase-8 is present and functional. Our data favor a model wherein caspase-8 activation and auto-processing downstream of IKK blockade restrain necroptosis, as *Casp8^D387A/D387A^* BMDMs undergo rapid RIPK3 and MLKL phosphorylation, and the cell death that occurs in *Casp8^D387A/D387A^* BMDMs is entirely MLKL- and RIPK3 kinase–dependent. RIPK3/MLKL-induced programmed necrosis also activates NLRP3, presumably via potassium efflux, thereby providing another route to caspase-1 engagement during *Yersinia* infection, even when caspase-8 cannot be activated. Thus, the coupling of NLRP3 activation to multiple types of lytic pores indicates an important role for backup mechanisms to ensure IL-1β releases and inflammation when immune signaling is inhibited or blocked by pathogen activity.

The absence of RIPK3 and MLKL phosphorylation during *Yersinia* infection of WT BMDMs together with the activation of caspase-8 before assembly of ASC specks and detectable caspase-1 activation suggests that distinct apoptotic and pyroptotic cell death complexes are activated sequentially during *Yersinia* infection. Caspase-1 is processed in a caspase-8–dependent manner even in *Asc^−/−^* or NLRP3-inhibited BMDMs, indicating that its initial activation likely occurs within the caspase-8–containing complex IIa. Critically, while active caspase-8 is found in punctate regions distributed throughout the cell following *Yptb* infection, consistent with previous reports (*51*), we did not observe colocalization between this active caspase-8 and ASC specks. Last, the reduced frequency of ASC specks that we observed in the absence of either caspase-1 and GSDMD suggests that caspase-1 serves as the primary activator of GSDMD, which then enables NLRP3 inflammasome activation. Together, our study reveals new insight into mechanisms of caspase-8–dependent activation of caspase-1, as well as further uncovers how pyroptotic and apoptotic cell death pathways communicate with one another to mediate antimicrobial host defense.

## MATERIALS AND METHODS

### Cell culture and differentiation of BMDMs

BMDMs were isolated and differentiated as previously described (*25*, *26*), in adherence to the National Institutes of Health’s *Guide for the Care and Use of Laboratory Animals* and under the guidelines and approval from the University of Pennsylvania Institutional Animal Care and Use Committee (protocol no. 804523). Briefly, isolated bone marrow cells from 6- to 10-week-old male and female mice were grown at 37°C and 5% CO_2_ in 30% macrophage medium [30% L929 fibroblast supernatant and complete Dulbecco’s modified Eagle’s medium (DMEM)]. BMDMs were harvested in cold phosphate-buffered saline (PBS) on day 7 and replated in 10% macrophage medium onto tissue culture (TC)–treated plates or glass coverslips in TC-treated plates. Transduced iBMDMs from *Casp1^−/−^* mice containing either WT caspase-1, caspase-1 DEAD, or empty vector were previously described and provided by D. Monack (*33*). Primary *Casp1^mlt/mlt^* BMDMs were previously described (*36*). *Casp8^D387A/D387A^Mlkl^−/−^* BMDMs were previously described (*44*) and provided by D. Green and B. Tummers. HEK293T cells were grown in complete DMEM [supplemented with 10% fetal bovine serum (FBS), 10 mM Hepes, 10 mM sodium pyruvate, and 1% penicillin/streptomycin] and maintained in a 37°C incubator with 5% CO_2_.

### Bacterial culture and in vitro infections

Bacterial strains: *Yptb* strain IP2666 (*63*), *Yptb* ΔYopJ (*64*), and *Salmonella enterica* serovar Typhimurium strain SL1344 (*65*) were all grown as previously described (*25*). Briefly, bacteria were grown with aeration and appropriate antibiotics at 28°C (*Yptb*, irgasan) or 37°C (*Salmonella*, streptomycin). *Yptb* strains were induced before infection by diluting stationary phase overnight cultures 1:40 in 3 ml of inducing medium[2× Yeast Extract Tryptone (YT) broth, 20 mM sodium oxalate, and 20 mM MgCl_2_]. Cultures were grown at 28°C for 1 hour and shifted to 37°C for 2 hours with aeration. *Salmonella* strains were induced before infection by diluting the overnight culture 1:40 in 3 ml of inducing medium (LB broth and 300 mM NaCl) and grown standing for 3 hours at 37°C. Bacterial growth was measured by absorbance at optical density at 600 nm (OD_600_) using a spectrophotometer. Bacteria were pelleted, washed, and resuspended in DMEM or serum-free medium for infection. In vitro infections were performed at multiplicity of infection of 20 unless otherwise noted. Gentamycin (100 μg/ml) was added 1 hour after infection for all infections.

### LDH cytotoxicity assay and ELISA

Triplicate wells of BMDMs were seeded in TC-treated 96-well plates. BMDMs were infected with indicated bacterial strains as indicated above. BMDMs were primed with LPS (100 ng/ml) for 3 hours followed by 2.5 mM ATP treatment or 5 hours of 10 μM IKKi (BMS-345541, Sigma-Aldrich) treatment. BMDMs were primed with Pam3CSK4 (400 ng/ml) overnight. BMDMs were treated with 1 μM GSK′872 (InvivoGen), 1 μM MCC950 (Tocris), and 60 μM Necrostatin-1 (InvivoGen) for 30 min, 1 hour, and 1 hour before infection, respectively. Gentamycin (100 μg/ml) was added 1 hour after treatment to all infectious experimental conditions. At indicated time points, plates were spun down at 250*g*, and supernatants were harvested. Supernatants were combined with LDH substrate and buffer (Sigma-Aldrich) according to the manufacturer’s instructions and incubated in the dark for 35 min. Plates were read on a spectrophotometer at 490 nm. Percent cytotoxicity was calculated by background subtraction and normalizing to maximal cell death (1% Triton X-100). To assess IL-1β release, supernatants were diluted fourfold and applied to Immulon enzyme-linked immunosorbent assay (ELISA) plates (ImmunoChemistry Technologies) pre-coated with anti–IL-1β capture antibody (eBioscience). Following blocking [1% bovine serum albumin (BSA) in 1× PBS], plates were incubated with biotin-linked anti–IL-1β detection antibody (R&D Systems, 1:1000), followed by horseradish peroxidase (HRP)–conjugated streptavidin. As readout for IL-1β levels, peroxidase enzymatic activity was determined by exposure to *o*-phenylenediamine hydrochloride (Sigma-Aldrich) in citric acid buffer. Reactions were stopped with sulfuric acid, and absorbance values were read at 490 nm and normalized to mock-transfected cells (negative control).

### HEK293T transfections

Mammalian expression plasmids containing indicated DNA constructs were transfected into HEK293T cells using Lipofectamine 2000 (Thermo Fisher Scientific) at 1:1 ratio (w/w DNA:Lipofectamine) in Opti-MEM (Gibco). Medium was changed to complete DMEM (10% v/v FBS) 6 hours after transfection. Twenty-four hours after transfection, cells were treated with 1 μM AP20187 (dimerizer, ApexBio) in serum-free DMEM for 6 hours in a humidified incubator at 37°C and 5% CO_2_ before subsequent analysis.

### Western blotting

BMDMs were seeded in TC-treated 24-well plates (3.0 × 10^5^ cells per well). HEK293T cells were seeded in poly-l-lysine–coated TC-treated 24-well plates (2.0 × 10^5^ cells per well) and transiently transfected with appropriate gene constructs as described above. Following infection or treatment in serum-free medium, supernatants were harvested, and trichloroacetic acid (TCA) was precipitated overnight at 4°C. Precipitated proteins were pelleted and washed with acetone. The pellet was resuspended in 5× sample buffer (125 mM tris, 10% SDS, 50% glycerol, 0.06% bromophenol blue, and 1% β-mercaptoethanol). BMDMs were lysed in lysis buffer [20 mM Hepes, 150 mM NaCl, 10% glycerol, 1% Triton X-100, and 1 mM EDTA (pH 7.5] plus 1× complete protease inhibitor cocktail and 1× sample buffer (25 mM tris, 2% SDS, 10% glycerol, 0.012% bromophenol blue, and 0.2% β-mercaptoethanol). Lysates and supernatants were boiled and centrifuged at full speed for 5 min, were run on 4 to 12% polyacrylamide gels, and transferred to polyvinylidene difluoride membrane. Membranes were immunoblotted using the following primary antibodies: β-actin (Sigma-Aldrich, 1:5000), caspase-1 (gift from V. Dixit, Genentech, 1:500), caspase-8 (Enzo, 1:1000), cleaved caspase-8 (Cell Signaling Technology, 1:1000) GSDMD (Abcam, 1:1000), and IL-1β (R&D Systems, 1:1000). Species specific HRP-conjugated secondary antibodies were used for each antibody (1:5000). Membranes were developed using Pierce ECL Plus and SuperSignal West Femto Maximum Sensitivity Substrate (Thermo Fisher Scientific) according to the manufacturer’s instructions. Western blot time courses were performed in parallel with cytotoxicity assays to accurately interpret protein release before and after overt cell death.

### Bioactive IL-1 reporter assay

Active IL-1β released from infected BMDMs was detected using HEK-Blue IL-1β (InvivoGen) reporter cells according to manufacturer’s instructions (InvivoGen). HEK-Blue IL-1β reporter cells express the IL-1R and encode secreted embryonic alkaline phosphatase (SEAP) driven by a nuclear factor κB promoter. This leads to IL-1β dose-dependent release of SEAP, which is quantified with the Quanti-Blue reagent (InvivoGen) and measuring the absorbance at OD_620_. HEK-Blue cells were seeded in 96-well plates at 5 × 10^4^ cells per well in DMEM with l-glutamine and 10% FBS to a total volume of 150 μl. Supernatant (50 μl) from control or treated mouse BMDMs were added, and the samples were incubated overnight at 37C in a 5% CO_2_ incubator. Supernatant (20 μl) was mixed with 180 μl of the Quanti-Blue solution and incubated at room temperature (RT) for 1 hour, and absorbance at OD_620_ was read on a Cytation 5 plate reader (BioTek). Recombinant murine IL-1β (PeproTech, 211-11B) was used to generate a standard curve to calculate absolute levels of active IL-1β present in supernatants.

### Fluorescence and confocal microscopy

BMDMs were seeded on circular glass coverslips (Thorlabs, no. CG15NH) and allowed to adhere overnight. Cells were then infected or transfected with the indicated DNA constructs (HEK293T cells). At the indicated time points, cells were fixed with 4% paraformaldehyde for 15 min, permeabilized with 0.2% Triton X-100 for 10 min, and blocked with 5% BSA for 1 to 2 hours. BMDMs were stained for cleaved caspase-8 (1:1000; Cell Signaling Technology, no. 8592S) or ASC (1:160; Millipore, no. 04-147) overnight at 4°C, Alexa Fluor 647–conjugated anti-rabbit (1:1000) and Alexa Fluor 488–conjugated anti-mouse (1:1000) at RT for 1 hour, and Hoechst at RT for 30 min. Cells were labeled with CellTracker Deep Red (Thermo Fisher Scientific) at a 0.1 μM concentration in serum-free medium for 30 min before seeding. Live/Dead Fixable dye (Invitrogen) was added to infected cells 4 hours after infection according to the manufacturer’s instructions. Cells were mounted on glass slides with Fluoromount-G (SouthernBiotech). Slides were imaged using either a Leica SP5-FLIM inverted confocal microscope or a Leica DM600 widefield microscope with a single **z**-plane taken per field. Lasers were optimized for green fluorescent protein (green), Cyanine 5 (Cy5, far-red), citrine (yellow), and 4′,6-diamidino-2-phenylindole (blue). Scale bar, 15 μm for all images.

### Image quantification and analysis

Each experiment was conducted in three technical replicates. Within each replicate, 20 to 30 fields of view were analyzed, with 80 to 200 cells (BMDMs) per field of view. Specks were defined as distinct high-fluorescence perinuclear clusters of citrine or Alexa Fluor 488 signal. Speck formation frequency was determined as the percentage of citrine-expressing cells that contained one or more specks, using custom macros from ImageJ. Percent of dead cells was determined as percentage of cells that stained Live/Dead positive relative to the total number of cells.

### Statistical analysis

Data were graphed and analyzed using GraphPad Prism. Mean values (± SEM) were compared across triplicate conditions, and *P* values were determined using the appropriate test and are indicated in each figure legend. Studies were conducted without blinding or randomization. Values of *P* < 0.05 were considered statistically significant.
