# Supplementary material for: Distinct sequential death complexes regulate pyroptosis and IL-1β release in response to Yersinia blockade of immune signaling
Source: Sci Adv. 2024 Jul 26;10(30):eadl3629. doi: 10.1126/sciadv.adl3629 (PMC11277400; doi:10.1126/sciadv.adl3629)
Supplement: Supplementary file 1 — Figs. S1 to S4 [file sciadv.adl3629_sm.pdf]

Science Advances
AAAS

Supplementary Materials for

Distinct sequential death complexes regulate pyroptosis and IL-1β release in response to *Yersinia* blockade of immune signaling

Ronit Schwartz Wertman *et al.*

Corresponding author: Igor E. Brodsky, ibrodsky@vet.upenn.edu

Sci. Adv. **10**, eadl3629 (2024)
DOI: 10.1126/sciadv.adl3629

This PDF file includes:

Figs. S1 to S4

Supplementary Materials

Figure S1 (related to Figure 2)- Caspase-1 catalytic activity is required for cell death during *Stm* infection: (S1A) iC57BL/6, *iCasp1*$^{-/-}$ + *Casp1*^{WT}, *iCasp1*$^{-/-}$ + *Casp1*^{DEAD}, and *iCasp1*$^{-/-}$ + *EV* iBMDMs were infected with WT *Stm*. Percent cytotoxicity was measured 4 hours post-infection. (S1B) C57BL/6, *Casp1*$^{mlt/mlt}$ BMDMs were infected with WT *Stm*. Percent cytotoxicity was measured 4 hours post-infection. (S1C) C57BL/6, *Casp1*$^{mlt/mlt}$ BMDMs were infected with WT *Yptb*. Release of IL-12 into the supernatant was measured by ELISA at 4 hours post-infection. (S1D) Lysates collected 3 hours post-infection were immunoblotted for caspase-3, GSDME, and β-actin. ns – not significant. ****$p < 0.0001$ by two-way ANOVA. Error bars represent the mean +/- SEM of triplicate wells and are representative of three independent experiments.

Figure S2 (related to Figure 3)- Individual cells sequentially activate pyroptosis downstream of necroptosis-mediated MLKL pore formation: (S2A) C57BL/6, *Casp8*$^{D387A/D397A}$, and *Casp8*$^{D387A/D387A}$*Mlkl*$^{-/-}$ BMDMs were treated with GSK'872, MCC950, or vehicle control and were infected with WT *Yptb*. Lysates collected 3 hours post-infection were immunoblotted for caspase-3, GSDME, and β-actin. (S2B-C) Unlabeled *Casp8*$^{D387A/D397A}$ and CellTracker-labeled *Casp8*$^{D387A/D387A}$*Mlkl*$^{-/-}$ BMDMs were individually cultured or co-cultured and were left uninfected or infected with WT *Yptb*. Live/Dead dye was added to the culture medium 3.5 hours post-infection for 30 min. (S2D) Percent dead cells was analyzed and quantified 4 hours post-infection via fluorescence microscopy. (S2E) CellTracker-labeled *Casp8*$^{D387A/D397A}$ were individually cultured and were infected with WT *Yptb*. ****$p < 0.0001$ by two-way ANOVA. Error bars represent the mean +/- SEM and are representative of three independent experiments.

Figure S3 (related to Figure 4)- Apoptotic caspases do not contribute to *Yptb*-induced pyroptosis: (S3A) C57BL/6 ASC-citrine BMDMs were primed with LPS followed by ATP treatment. ASC speck formation was analyzed 1-hour post ATP treatment. (S3B) C57BL/6, and *Asc*$^{-/-}$ BMDMs were infected with WT *Yptb*. ASC speck formation was analyzed 4 hours post-infection. (S3C) C57BL/6, *Asc*$^{-/-}$, and *Nlrp3*$^{-/-}$ BMDMs were treated with MCC950 or vehicle control and were infected with WT *Yptb*. Percent cytotoxicity was measured 4 hours post-infection. (S3D) C57BL/6 and *Panx1*$^{-/-}$ BMDMs were treated with MCC950 or vehicle control and were infected with WT *Yptb*. Percent cytotoxicity was measured 4 hours post-infection. (S3E) Release of IL-1β into the supernatant was measured by ELISA at 4 hours post infection. (S3F) C57BL/6 and *Asc*$^{-/-}$ BMDMs were infected with WT *Yptb*. Lysates collected 3 hours post-infection were immunoblotted for caspase-3, GSDME, and β-actin. (S3G) C57BL/6 and *Casp3*$^{fl/fl}$*Casp7*$^{-/-}$ *Tie2Cre*$^{+}$ BMDMs were infected with either WT or Δ*yopJ Yptb*. Percent cytotoxicity was measured 4 hours post-infection. ns — not significant. Error bars represent the mean +/- SEM of triplicate wells and are representative of three independent experiments.

Figure S4 (related to Figure 5)- Absence of casp1/11 results in reduced ASC speck formation: (S3A) C57BL/6, and *Casp8*$^{-/-}$*Ripk3*$^{-/-}$ BMDMs were infected with WT *Yptb*. Caspase-8 cleavage was analyzed 4 hours post-infection. (S3B) 1 hour prior to infection *Casp1/11*$^{-/-}$ ASC-citrine BMDMs were treated with MCC950, Nec-1, or vehicle control and were infected with WT *Yptb*. Caspase-8 cleavage and ASC speck formation were analyzed at 4 hours post-infection. (S3C) Quantification of percent of cells with ASC specks for all conditions. (S3D) *Casp1/11*$^{-/-}$ C57BL/6 ASC-citrine BMDMs were primed with LPS followed by ATP treatment. ASC speck formation was analyzed 1-hour post ATP treatment. **** $p < 0.0001$ by two-way ANOVA. Error bars

represent the mean +/- SEM of triplicate wells and are representative of three independent experiments.

Figure S1

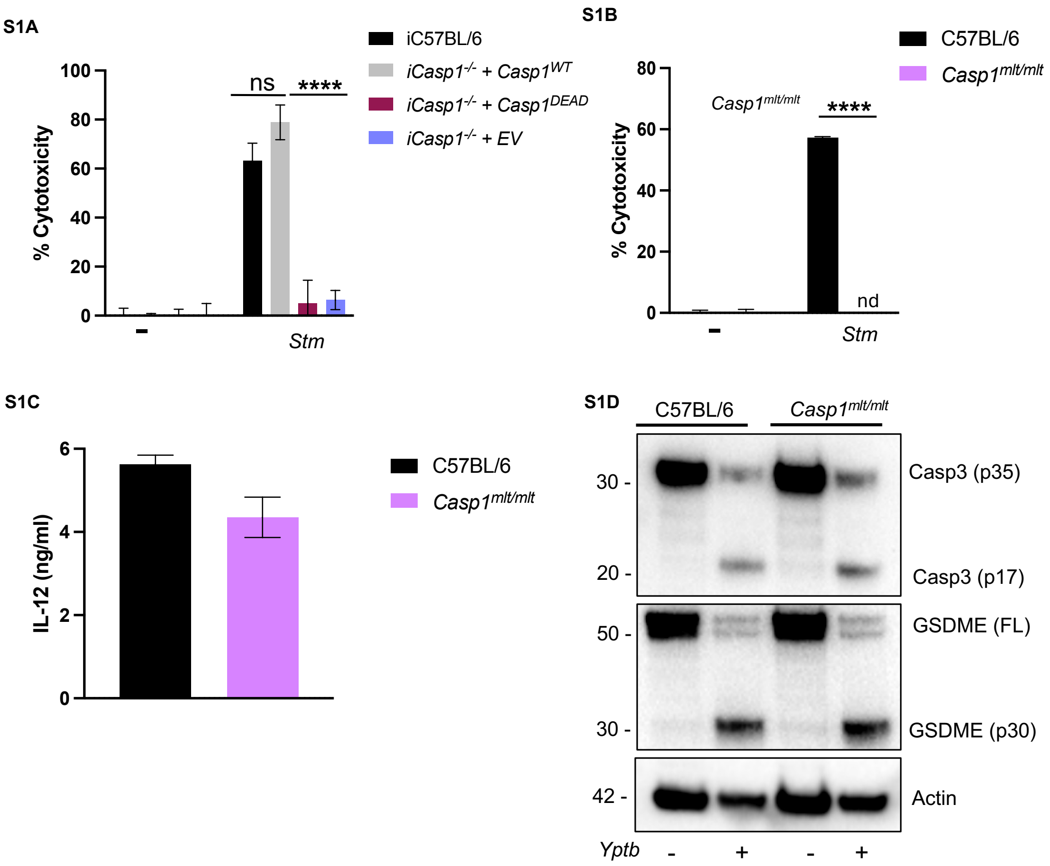
S1A
iC57BL/6
iCasp1-/- + Casp1WT
iCasp1-/- + Casp1DEAD
iCasp1-/- + EV
% Cytotoxicity
100
80
60
40
20
0
ns
****
-
Stm
S1B
C57BL/6
Casp1mlt/mlt
Casp1mlt/mlt
****
% Cytotoxicity
80
60
40
20
0
nd
-
Stm
S1C
C57BL/6
Casp1mlt/mlt
IL-12 (ng/ml)
6
4
2
0
S1D
C57BL/6
Casp1mlt/mlt
30 -
20 -
Casp3 (p35)
Casp3 (p17)
50 -
30 -
GSDME (FL)
GSDME (p30)
42 -
Actin
Yptb
-
+
-
+

Figure S2

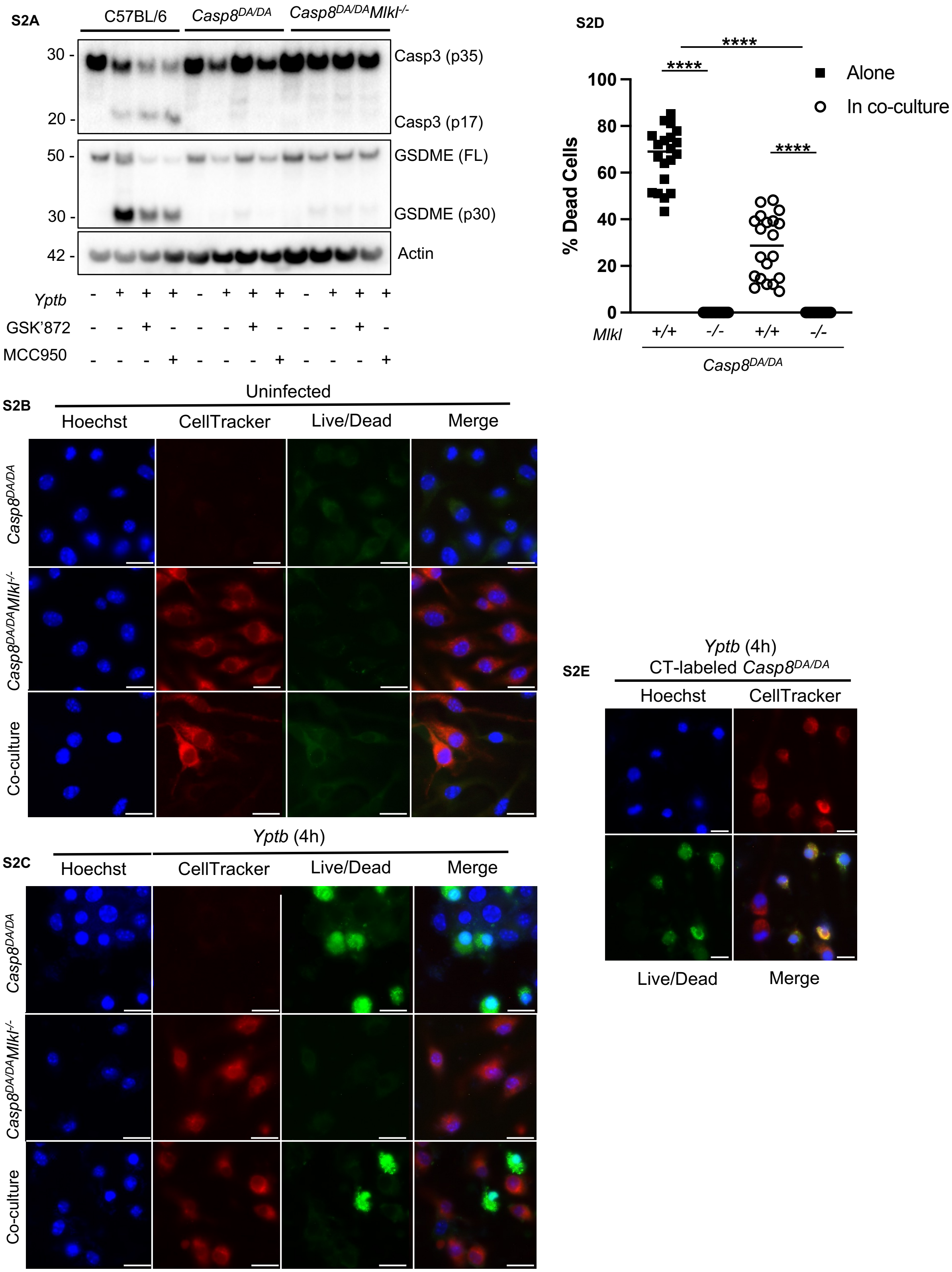

S2A
C57BL/6
Casp8DA/DA
Casp8DA/DAMlkl-/-
30 -
20 -
50 -
30 -
42 -
Casp3 (p35)
Casp3 (p17)
GSDME (FL)
GSDME (p30)
Actin
Yptb
GSK'872
MCC950
S2D
% Dead Cells
Alone
In co-culture
Mlkl
+/+
-/-
Casp8DA/DA
****
S2B
Uninfected
Hoechst
CellTracker
Live/Dead
Merge
Co-culture
S2C
Yptb (4h)
S2E
CT-labeled Casp8DA/DA

Figure S3

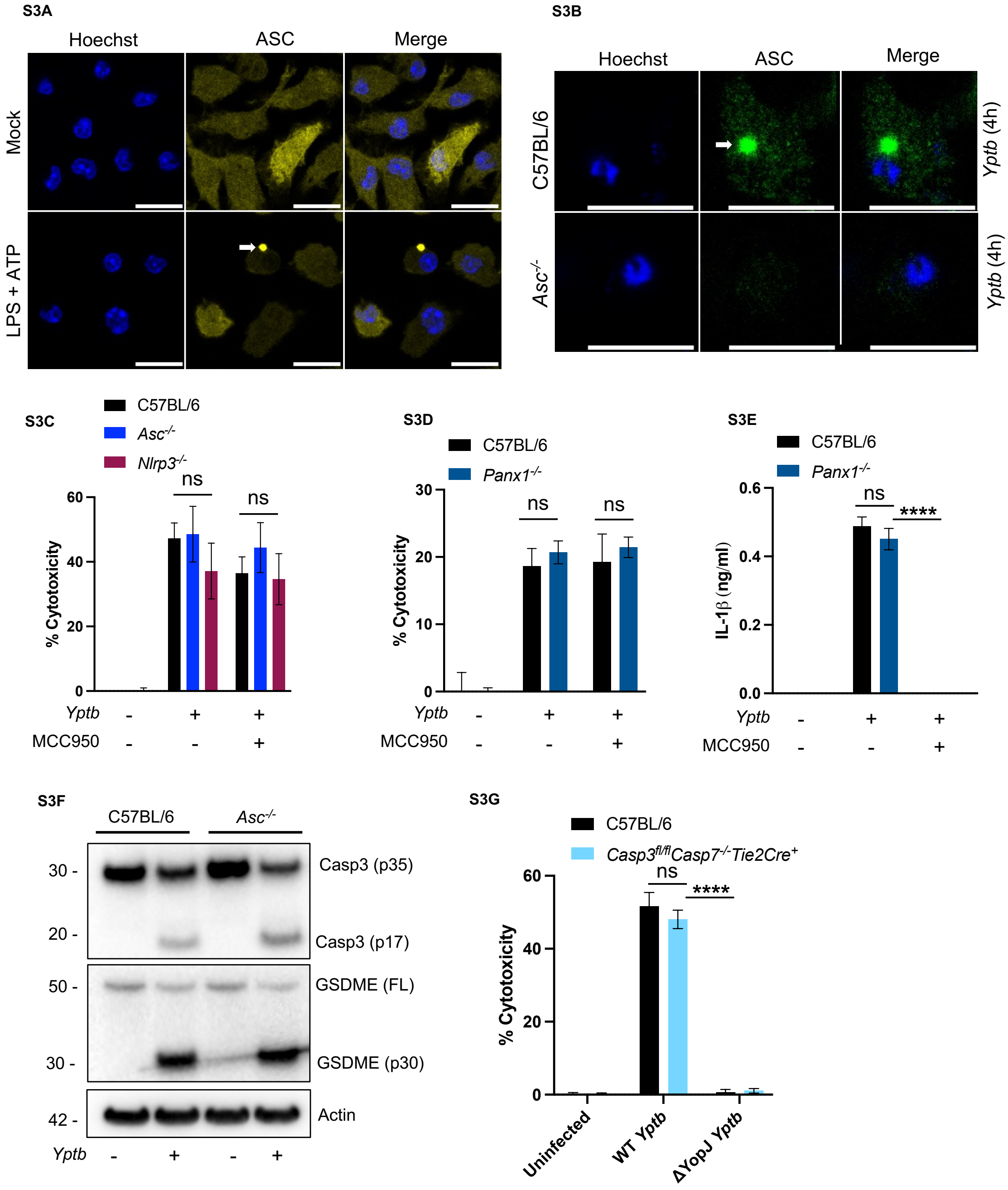

S3A
Hoechst
ASC
Merge
Mock
LPS + ATP
S3B
C57BL/6
Asc-/-
Yptb (4h)
S3C
C57BL/6
Asc-/-
Nlrp3-/-
ns
% Cytotoxicity
Yptb
MCC950
S3D
Panx1-/-
S3E
IL-1β (ng/ml)
****
S3F
Casp3 (p35)
Casp3 (p17)
GSDME (FL)
GSDME (p30)
Actin
S3G
Casp3fl/flCasp7-/-Tie2Cre+
Uninfected
WT Yptb
ΔYopJ Yptb

Figure S4

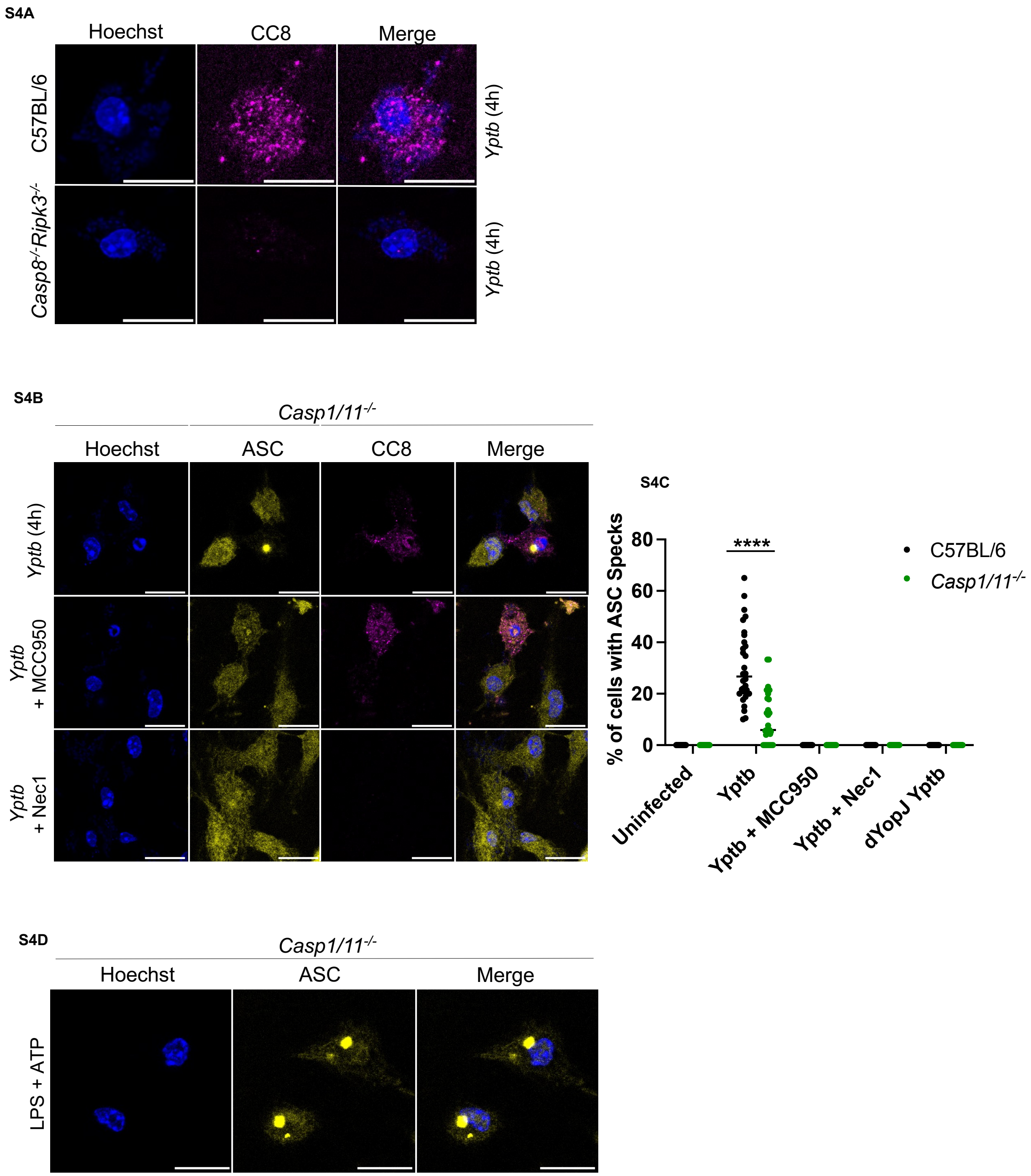
S4A
Hoechst
CC8
Merge
C57BL/6
Casp8-/-Ripk3-/-
Yptb (4h)
Yptb (4h)
S4B
Casp1/11-/-
Hoechst
ASC
CC8
Merge
Yptb (4h)
Yptb + MCC950
Yptb + Nec1
S4C
% of cells with ASC Specks
80
60
40
20
0
****
C57BL/6
Casp1/11-/-
Uninfected
Yptb
Yptb + MCC950
Yptb + Nec1
dYopJ Yptb
S4D
Casp1/11-/-
Hoechst
ASC
Merge
LPS + ATP
